# Evaluating the stability of nursery-established arbuscular mycorrhizal fungal associations in apple rootstocks

**DOI:** 10.1128/aem.01937-24

**Published:** 2024-12-10

**Authors:** Huiting Zhang, Wanyan Wang, Loren Honnas, Mark Mazzola, Tracey Somera

**Affiliations:** 1 Department of Horticulture, Washington State University 271508 , Pullman, Washington, USA; 2 Ecosystem Science and Management, Pennsylvania State University 142849 , State College, Pennsylvania, USA; 3 USDA-ARS Tree Fruit Research Laboratory 57748 , Wenatchee, Washington, USA; 4 Department of Plant Pathology, Stellenbosch University 26697 , Matieland, South Africa; Royal Botanic Gardens, Surrey, United Kingdom

**Keywords:** arbuscular mycorrhizal fungi, *Malus domestica*, rootstock genotype, colonization efficacy

## Abstract

Arbuscular mycorrhizal fungi (AMF) are promoted as commercial bioinoculants for sustainable agriculture. Little is known, however, about the survival of AMF inoculants in soil and their impacts on native or pre-established AMF communities in root tissue. The current study was designed to assess the stability of pre-existing/nursery-derived AMF in apple rootstocks after being planted into soil containing a known community of AMF with a limited number of species. Root-associated endophytic communities (bacteria and fungi) are known to differ depending on apple rootstock genotype. Thus, an additional aim of this study was to explore the effect of apple rootstock genotype on AMF community structure. A greenhouse experiment was conducted in which a variety of apple rootstock genotypes (G.890, G.935, M.26, and M.7) were inoculated with a commercially available, multi-species AMF consortium. Nursery-derived AMF communities were sequenced, and changes to AMF community structure following cultivation in pasteurized soil (inoculated and non-inoculated) were assessed using a Glomeromycota-specific phylogenetic tree, which included 91 different AMF species from 24 genera. Results show that inoculant colonization potential was limited and that apple rootstocks serve as a significant source of inoculum from the nursery where they are produced. Rootstocks established relationships with introduced AMF in a genotype-specific manner. Regardless of colonization success, however, the inoculant caused alterations to the resident AMF communities of both Geneva and Malling rootstocks, particularly low abundance taxa. In addition, phylogeny-based analysis revealed a unique, well-supported clade of unknown taxonomy, highlighting the importance of using phylogenetic-based classification for accurate characterization of AMF communities.

**IMPORTANCE:**

Understanding the impacts of introduced AMF on residential AMF communities is essential to improving plant productivity in nursery and orchard systems. In general, there is a dearth of data on the interactions of commercial AMF inoculants with pre-established AMF communities living in symbiosis with the host plant. The interplay between apple rootstock genotype and the endophytic root microbiome is also an area where more research is needed. This study demonstrates the potential for nursery-established AMF associations to be maintained when transplanted into the field. In addition to providing insight into rootstock/AMF associations, our study calls attention to the current issues attendant with relying on web-based databases for determining AMF identity. The use of phylogenetic tools represents one possible solution and may be of value to industry practitioners in terms of improving product composition and consistency.

## INTRODUCTION

Endophytic microorganisms living inside plant tissues often play very specific and crucial roles in promoting the health and growth of their host plant. For example, most plants in the legume family (i.e., Fabaceae) recruit nitrogen-fixing bacteria (rhizobia) to live inside nodules on their roots (1). Like rhizobia, arbuscular mycorrhizal fungi (AMF) belonging to the phylum Glomeromycota are another group of soilborne endosymbionts generally considered to be beneficial (2). AMF form diverse relationships with a host of land plants (roughly 80% of all species) (3, 4). This speaks to the importance of these fungi in the health and functioning of entire terrestrial ecosystems, including agroecosystems. Mycorrhizal hyphal strands (3–7 μm) are considerably smaller in diameter than fine roots (<1–2 mm) or even root hairs (5–20 μm) (5, 6), enabling them to extend into otherwise inaccessible soil patches. This allows AMF to scavenge resources necessary for plant growth including: phosphorous (7–9), nitrogen (10, 11), and water (12, 13). In addition to improving plant resource availability, AMF improve soil structure (14), carbon content (15, 16), and hydraulic properties (17) while occupying a niche in the root that might otherwise be filled by disease-causing soilborne organisms (18). Colonization by mycorrhizal fungi has also been shown to reduce pest and pathogen pressure above ground in a number of crops (including apple) (19–23).

Although mycorrhizal associations have been shown to improve productivity/yield in a diversity of cropping systems (24–29), including fruit tree orchards (30, 31), structuring AMF–plant relationships to improve productivity and sustainability in agroecosystems is inherently difficult. This is because soil tillage (32), soil compaction (33), high soil phosphorous content, and the use of systemic fungicides can all negatively impact AMF colonization rates (34, 35). As a result, little information exists on the survival/effectiveness of AMF inoculants in the soil and their impacts on native or pre-established AMF communities in root tissue. Within the limited body of research that has been done, results are often conflicting, and inoculated isolates are not always distinguished from native AMF. A review of several studies using the inoculant *Rhizophagus irregularis* illustrates the problems. For example, in a study by Renaut et al. (36) inoculation of field plots after sowing corn, soybean, or wheat seeds did not alter the composition of the indigenous AMF community in any of the crops (36). Native and inoculated strains of *R. irregularis* could not be distinguished (36). In a different study by Buysens et al. (37), *R. irregularis* inoculant (applied at planting) was successfully traced in the field (37). Compared with native *R. irregularis* strains, the inoculant was detected at low levels in very few plants. In another study, however, inoculation at sowing with a commercially available strain of *R. irregularis* reduced the diversity of indigenous AMF detected in pea roots relative to non-inoculated controls. Here, the commercial inoculant appeared to be most effective at displacing closely related taxa (e.g., indigenous *Rhizophagus* spp.) (38). Similarly, in yet another study, pre-inoculation of plants with *R. irregularis* was found to suppress colonization by native AMF, especially those of the same species (39). Finally, in the study by Islam et al. (38), differences in AMF community structure between inoculated vs control treatments lessened over time (i.e., three growing seasons), suggesting that introduced AMF taxa may not persist long term among locally adapted AMF communities (38).

In addition to environmental compatibility (i.e., the ability of a given taxon to thrive in the habitat in question), AMF community assembly is shaped by a number of other factors. In apple, there is evidence suggesting a selective capacity of rootstock genotype on the fungal endophytic microbiome (40). Certain rootstock genotypes may be more effectively colonized by or compatible with particular AMF species than with others (41). The current study was specifically designed to assess the ability of a known community of AMF with a limited number of species (i.e., the AMF inoculant) to compete with pre-existing/nursery-derived AMF contained in apple roots (without the confounding factor of site). To further investigate the selective capacity of rootstock genotype on the AMF community, a variety of commercially available apple rootstock genotypes (G.890, G.935, M.7, and M.26) were used. It was hypothesized that the commercial AMF inoculant would survive and differentially colonize new root tissue depending on the apple rootstock genotype utilized, altering pre-established/nursery-derived AMF communities.

In our investigation, rootstocks were cultivated in pasteurized potting soil with or without the commercial AMF consortium. After 1 month, plants were harvested, and new root tissue was collected for DNA extraction. The subsequent changes to the nursery-derived AMF community (established prior to planting) were then characterized using the Glomeromycota-specific primer set AML1/2, which targets the V3–V4–V5 regions of the nuclear 18S rRNA gene (42). It should be noted that the nuclear ribosomal internal transcribed spacer (ITS) region is the molecular marker used by the scientific community to identify fungi. Although this region can accurately detect AMF, it is not suitable for lower-level taxonomic discrimination (43, 44). Instead, the nuclear rDNA region (in particular the 18S rRNA gene) is widely used for characterizing AMF diversity. Over the last few decades, the available nuclear rDNA sequence data have grown considerably, enabling molecular studies of AMF communities. At present, however, there are no 18S rRNA databases for molecular identification of AMF that are actively curated. Therefore, as part of this study, a phylogenetic tree for Phylum Glomeromycota was constructed using over 140 high-quality (i.e., from well-identified AMF cultures) 18S rRNA gene sequences (43, 44). This phylogenetic tree (which included 91 different AMF species from 24 different genera) was used to accurately assign high throughput sequencing data to the species level.

## MATERIALS AND METHODS

### Selection and preparation of inoculum

The multi-species AMF formulation used in this study (Mycorrhizal Applications; Grants Pass, OR) was desirable because, compared with other commercially available mixtures, it was purported to contain a diverse AMF consortium, including a number of ecologically relevant colonizers previously associated with apple. According to the manufacturer, the product contained the following five mycorrhizal fungal species: *Glomus clarum* (Order Glomerales); *Glomus monosporum* (Order Glomerales); *Septoglomus deserticola*, formerly *Glomus deserticola* (Order Glomerales); *Paraglomus brasilianum* (Order Archaeosporales); and *Gigaspora margarita* (Order Gigasporaceae), 132 ppg/g. The experiment was conducted using pasteurized potting soil (Sunshine Professional Growing Mix #1; Sun Gro Horticulture, Abbotsford, Canada). Soil was pasteurized at 80°C for 2 × 8-h cycles (with a cool-down/aseptic mixing step between cycles). The five-species consortia were hand-mixed into pasteurized potting soil prior to planting at the recommended field rate per the manufacturer’s instructions (500–600 propagules/tree; 4.5 g/tree). This application rate is referred to as “1×” in the figures and text below.

### Experimental design and planting

Rootstocks used in this study included G935, G.890, M.7, and M.26; 0.635 cm trunk diameter. All rootstocks were obtained in a single shipment from TRECO (Woodburn, OR, USA). For each rootstock × treatment combination, five replicates were planted into 2.7-L pots containing pasteurized potting mix with or without the AMF inoculum. Pots were arranged in a completely randomized design on the greenhouse bench. Immediately prior to planting, a small amount of fine root tissue (~2 g) was collected from various locations on the root system in order to obtain a representative sample of the “nursery-derived” AMF community. Root tissue was stored at −80°C until processing. Initial trunk diameter (cm) was measured at planting using a caliper. There were 40 experimental plants in total + additional “sentinel” plants for the different rootstock genotypes. Sentinel plants were used to verify that a sufficient amount of new root tissue had been produced before harvesting the experiment. Rootstocks were grown for a period of 4 weeks at 22°C–28°C under supplemental lighting to maintain a 16-h photoperiod and watered as needed; plants did not receive supplemental nutrients. At 4 weeks post-planting, the rootstocks were removed from pots, and new (white) root tissue was collected from each plant for DNA isolation. To avoid sampling root tissue that may have been present at the nursery, only white roots were collected. Plant growth characteristics were also measured at this time and included: trunk diameter (cm), total plant biomass (g), root volume (mL), and leader shoot length (cm).

### DNA extraction from root tissue

Prior to DNA extraction, all root tissues were thoroughly rinsed in tap water to remove any residual soil particles/debris and blotted dry. Cleaned roots were then ground to a fine powder in liquid nitrogen by using a sterile mortar/pestle. Because the focus of this study is on the root-associated AMF community, roots were not pre-sterilized (as is done for endophytes). In other words, DNA was extracted from root surface + endosphere. Ground root tissue was preserved at −80°C until molecular analysis. The DNeasy Plant Pro Kit (Qiagen, Valencia, CA, USA) was used according to the manufacturer’s instructions to isolate DNA from 50 mg of ground root tissue per plant.

### DNA extraction from soil

Because pasteurized potting mix represents an ablated soil microbiome (with no AMF species present), it was assumed that the commercial inoculum would be the main source of “new” AMF. In order to confirm the absence of AMF in pasteurized potting soil (prior to adding inoculum/planting), DNA was extracted from 3 × 10 g of pasteurized potting soil using the DNeasy PowerMax Soil Kit (Qiagen, Valencia, CA, USA). These samples were sequenced as described below.

### DNA extraction from commercial AMF inoculum

DNA was also directly extracted and sequenced from the commercial AMF mixture in order to confirm the species composition. AMF spores and hyphae were extracted from 125 mL of the granular formulation *via* wet sieving (500, 250, and 45 μm metal sieves) followed by sucrose-density gradient centrifugation (using 20% and 60% sucrose) as described by INVAM (https://invam.ku.edu/spore-extraction). DNA was extracted from the concentrated spore sample using the DNeasy Plant Pro Kit (Qiagen, Valencia, CA, USA) according to the manufacturer’s instructions. Two samples from separate DNA extractions were sequenced.

### 18S amplification and DNA sequencing

Prior to sequencing, all DNA samples were quantified using a Qubit Fluorometer (Thermo Scientific, Waltham, MA, USA), and sample integrity was verified as described by Van Horn et al. (40). Briefly, samples were subjected to PCR amplification of the fungal internal transcribed spacer (ITS) rRNA gene, followed by visualization on a 2% agarose gel. Amplicons were generated using the Glomeromycotan-specific primers AML1 (5′-ATC AAC TTT CGA TGG TAG GAT AGA-3′) and AML2 (5′-GAA CCC AAA CAC TTT GGT TTC C-3′) (42) and sequenced on a PacBio Sequel instrument (average reads per sample = 10,000) using the circular consensus sequencing (CCS) mode. PacBio “long-read” sequencing technology (45) was used because the AML1/2 primer set amplifies a relatively long section of the 18S (SSU) rRNA (amplicon size = ~800 bp). The CCS was used to generate high fidelity consensus sequences by correcting the stochastic errors generated in each round of sequencing. Sequencing results were obtained from the sequencing facility (Molecular Research, Shallowater, TX, USA), demultiplexed in house and then pre-processed using the DADA2 (v1.26.0) pipeline, which included quality filtering, trimming, and dereplicating (46). Dereplicated sequences were then used for sequencing error estimation, and the error model was used in denoising and chimera removal (46). The amount and percentage of reads that passed each step are listed in Table S1. The DADA2 algorithm was then used to assign amplicon sequence variants (ASVs) to the processed reads and to summarize the results into an ASV table in which each row represents an ASV and the number of ASVs observed in each sample (column) are listed. It is worth mentioning that many AMF species have a high-level of intra-genomic heterogeneity of ribosomal sequences (i.e., each spore can have dozens of sequence variants). This complicates the ability to accurately determine the number of species based on clustering sequences (44). In this study, CCS was used to generate ASVs differing by as little as one nucleotide, an approach that is appropriate for not lumping closely related species together. Raw sequencing data are located in the NCBI Sequence Read Archive (SRA) under NCBI BioProject ID PRJNA1124126.

### Relative abundance calculation

Prior to analysis of ASV read counts, data were edited to remove singletons and doubletons as well as non-Glomeromycotan ASVs. The relative abundance of each ASV was calculated per the total reads in each sample (Table S2). Heatmaps were generated using the python Seaborn heatmap package (47) with average abundance of the biological replicates as input.

### Phylogenetic analysis and taxonomy assignment of AMF ASVs

**Fig 1:**
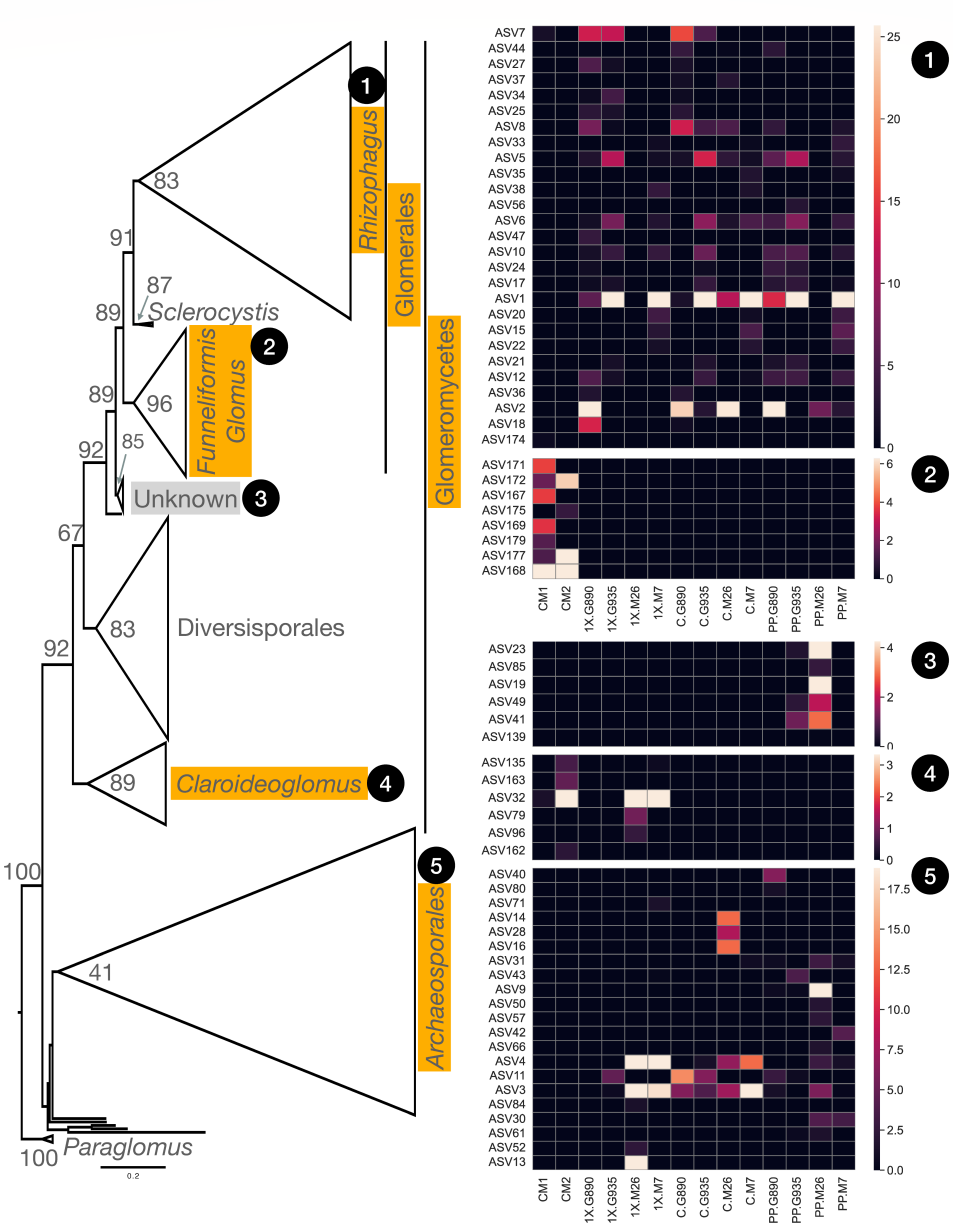
(Left) A simplified version of the full phylogenetic tree for Phylum Glomeromycota. Triangles represent major clades that have been collapsed in order to visualize tree structure. Triangle length represents maximum sequence divergence within a clade; triangle height represents the number of sequences in the clade. Numbers at each node represent bootstrap values. The scale bar indicates substitution rate. Phylogenetic trees were viewed and edited using FigTree v.1.4.4. Clades highlighted in orange contain amplicon sequence variants (ASVs) recovered from our sequencing analysis along with reference sequences. Clades highlighted in gray only contain ASVs from our sequencing analysis that are not taxonomically annotated (i.e., no reference sequences). Clades with no background contain only reference sequences. (Right) Heatmaps showing relative abundance of ASVs within the major clades shown on the left (labeled 1–5). Experimental treatments are listed along the x-axis of the heat map; CM (commercial mixture) =DNA extracted directly from the raw material; 1× = DNA extracted from rootstocks inoculated with CM; C (control) = DNA extracted from non-inoculated rootstocks; PP (pre-planting) = DNA extracted from roots prior to use in the experiment. The left y-axis lists ASVs identified in the experimental samples; the relative abundance of each ASV is depicted on the right y-axis along a color gradient (the lighter the color, the more abundant the ASV). Values represent the mean of 4–6 biological replicates for each treatment; CM1 and 2 represent individual samples.

Before performing phylogenetic reconstruction analyses, a total of 174 well-curated Glomeromycota 18S (SSU) rRNA reference sequences (which included 91 different species from 24 different genera) were gleaned from two different studies (43, 44) (summarized in Supplementary Files S1 and S2, respectively). AMF ASVs from this study were integrated with 1) all 174 sequences from the two reference sets or 2) with only the reference sequences from Krüger et al. (43), creating two sets of sequences for downstream analyses. Analysis of our AMF ASV with only the Stefani et al. (44) data set was not performed due to the small number of reference sequences (number of sequences = 28) provided by Stefani et al. (44). Next, nucleotide sequence alignments of the two sets of sequences were executed using the GeneFamilyAligner tool from PlantTribes2 (48) with the MAFFT algorithm. Sequences beyond the AML1/2 primer binding region were trimmed from the alignment using Geneious (v. 9.0.5) (49). Maximum likelihood phylogenetic trees were computed using the command line version of IQtree2 (50) with automated substitution model selection enabled, 2,000 ultra-fast bootstrapping and bnni refinement selected. *Paraglomus* was used as an outgroup as it represents the most basal glomeromycotan branch. Phylogenetic trees inferred from the two sequences sets have similar topology; however, the tree with sequences from both reference papers has an overall lower bootstrap support, likely due to a few sequences with divergent nucleotides in regions highly conserved in other sequences. As a result, the tree inferred with our AMF ASV plus reference sequences from Krüger et al., (43) was selected for downstream analysis (Fig. S1: full tree file). Based on information from Krüger et al. (43), this tree included 21 sequences from type/ex-type cultures. The tree supported five major clades (some are at order level, some family level). The simplified version of the tree showing these five clades was created using FigTree v.1.4.4 (Fig. 1) (51). Interestingly, an ASV clade with long branch length was observed in the phylogenetic tree. Further investigation into the sequence alignment showed that those ASVs contain extra nucleotides not shared with sequences outside this clade (Fig. S2A and B). In order to test whether long branches were caused by regions that did not align with other sequences, different alignment trimming stringencies were tested: removal of sites with 90% + gaps, 50% + gaps, 10% + gaps, or 0% + gap (in which any sites with gaps are removed). Additional phylogenetic trees were inferred using the same method described above with the trimmed alignments. The trimming did not affect the tree topology or branch length (except the most rigorously trimmed tree; 0% + gap). The bootstraps were also stable. However, the tree with the highest overall bootstrap value did not receive any post-alignment trimming and was selected for taxonomy assignment (Fig. S1). Taxonomy was assigned to ASVs by identifying monophyletic groups housing ASV and curated reference sequences. For instance, ASVs 177, 178, 179, and 166 were annotated as *Funneliformis coronatus* because they were found in the same monophyletic group with *F. coronatus* reference sequences; ASV 174 was annotated only to the genus level, *Rhizophagus*, as it fell between different *Rhizophagus* species from the reference.

### Statistical analyses

Significant differences between nursery-derived AMF communities and those existing after planting were assessed using relative abundance data via One-way analysis of similarities (ANOSIM) with the Bray–Curtis dissimilarity coefficient. For each rootstock genotype, significant differences in growth characteristics between AMF-inoculated and non-inoculated control treatments were assessed using Two-way analysis of variance (ANOVA) (with rootstock genotype and treatment as factors) and means were compared by Tukey’s multiple comparisons test (*P* ≥ 0.05). Growth data (increase in trunk diameter, shoot biomass, and root volume) were transformed prior to analysis (y = log(y)) and tested for normality. All transformed data sets passed Anderson–Darling, D’Agostino and Pearson, Shapiro–Wilk, and Kolmogorov–Smirnov normality tests. For each rootstock genotype, Mann–Whitney tests (Holm–Sidal method) were used to check for significant differences in root:shoot biomass ratios at harvest. Significant differences in the amount of AMF DNA detected in root tissue (as estimated from qPCR of total fungal DNA) were assessed using Two-way ANOVA followed by Tukey’s multiple comparisons test.

### Quantitative estimation of AMF in root tissue

In this study, because only frozen root tissue was available, the percentage of AMF colonization could not be directly evaluated *via* microscopy. Instead, AMF abundance was assessed in relation to the total amount of fungi present *via* a combination of methods. First, the fungal community was sequenced using the universal ITS1f (5′ CTTGGTCATTTAGAGGAAGTAA)/ITS2r (5′-GCTGCGTTCTTCATCGATGC) primer pair. Briefly, DNA extracted from root surface + endosphere (as described in Section 2.3) was sent to the sequencing facility (Molecular Research, Shallowater TX, USA) where it was PCR-amplified (prior to library preparation) and sequenced using an Illumina NovaSeq platform (20,000 reads per sample). Paired-end sequences were joined, and those <150 bp or with ambiguous base calls were removed. Sequences were quality filtered using a maximum expected error threshold of 1.0, dereplicated, and denoised. Final ASVs were taxonomically classified using BLASTn against a curated database derived from NCBI (www.ncbi.nlm.nih.gov). Prior to ASV read count analysis, data were edited to remove singletons and doubletons as well as any non-fungal reads. The abundance of Glomeromycota reads relative to total fungal reads in each sample was then calculated.

The absolute amount of fungal DNA present in these samples was also measured using a QuantStudio3 Real-Time PCR System with the NSI1 (5′-GAT TGA ATG GCT TAG TGA GG) (52) and 5.8S (5′-CGC TGC GTT CTT CAT CG) (53) primer pair. Run conditions were performed as described in Somera et al. (54). Purified genomic DNA from *Illonectria robusta* (isolate # 14–264) was used to generate the standard curve with a dilution range from 0.01 to 100 pg µL−1. All reactions were performed in triplicate, and each 96-well plate included a no-template control. Relative percentages of AMF were then transformed into absolute values using fungal DNA quantities.

## RESULTS

### Overview of PacBio sequencing statistics

A total of 640,087 AMF 18S rRNA sequence reads were obtained after cleaning/de-noising. The use of PacBio sequencing technology allowed for the generation of relatively long sequence reads of high quality, with sufficient information content for phylogenetic analysis (which is not the case with shorter amplicons; <300 bp). In this study, median read length ranged from 794 to 1,053 bp (Fig. S3). Rarefaction analyses indicated that the sequencing depth (10,000 reads per sample) sufficiently captured AMF diversity in the roots; the curves reached the asymptote around a depth of 7,000 (Fig. S4). A total of 180 AMF amplicon sequence variants (ASVs) was detected across all experimental samples.

### Phylogeny-based assessment of AMF community composition in apple rootstocks

In many studies, AMF taxonomy is determined using the AMF-specific reference sequence database MaarjAM (https://maarjam.ut.ee/) (55). However, as noted above, this database is no longer actively curated. In this study, tree-based (phylogenetic) taxonomic assignments of sequence data were used to assess AMF community structure in apple root tissue pre- and post-planting. The phylogenetic tree in this study (Fig. S1) shares the same major clades with trees presented in both references (Fig. S5), namely studies by Krüger et al. (43) and Stefani et al. (44). Our tree shared a more similar clade-to-clade relationship to the Krüger tree and resolved with high-confidence some unresolved relationships in the Kruger tree. Tree-based taxonomy assignment showed that experimental ASVs represented taxa from a handful of genera, including *Rhizophagus*, *Funneliformis*, *Claroideoglomus,* and *Ambispora/Archaeospora* (Fig. 1). Phylogenetic analysis was also consistent with previous studies in which *R. irregularis* and *R. intraradices* are well separated (56).

### Pre-planting (nursery-derived) AMF

**Fig 2:**
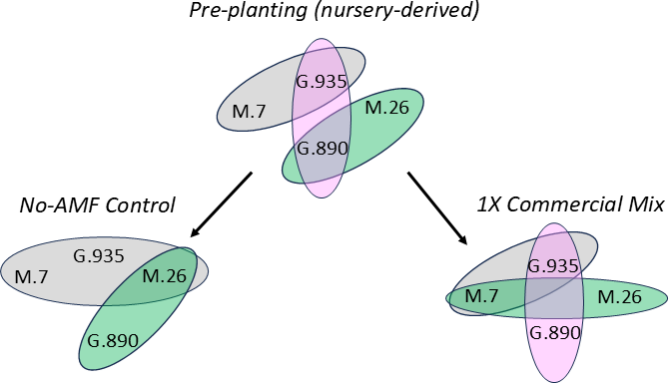
Venn diagrams reflecting how nursery-derived AMF communities diverged after planting into pasteurized potting mix (No-AMF Control; left) or into pasteurized potting mix containing the AMF product (1× Commercial Mix; right). AMF community composition was not significantly different between rootstock genotypes sharing the same color according to one-way ANOSIM of relative abundance data.

**Fig 3:**
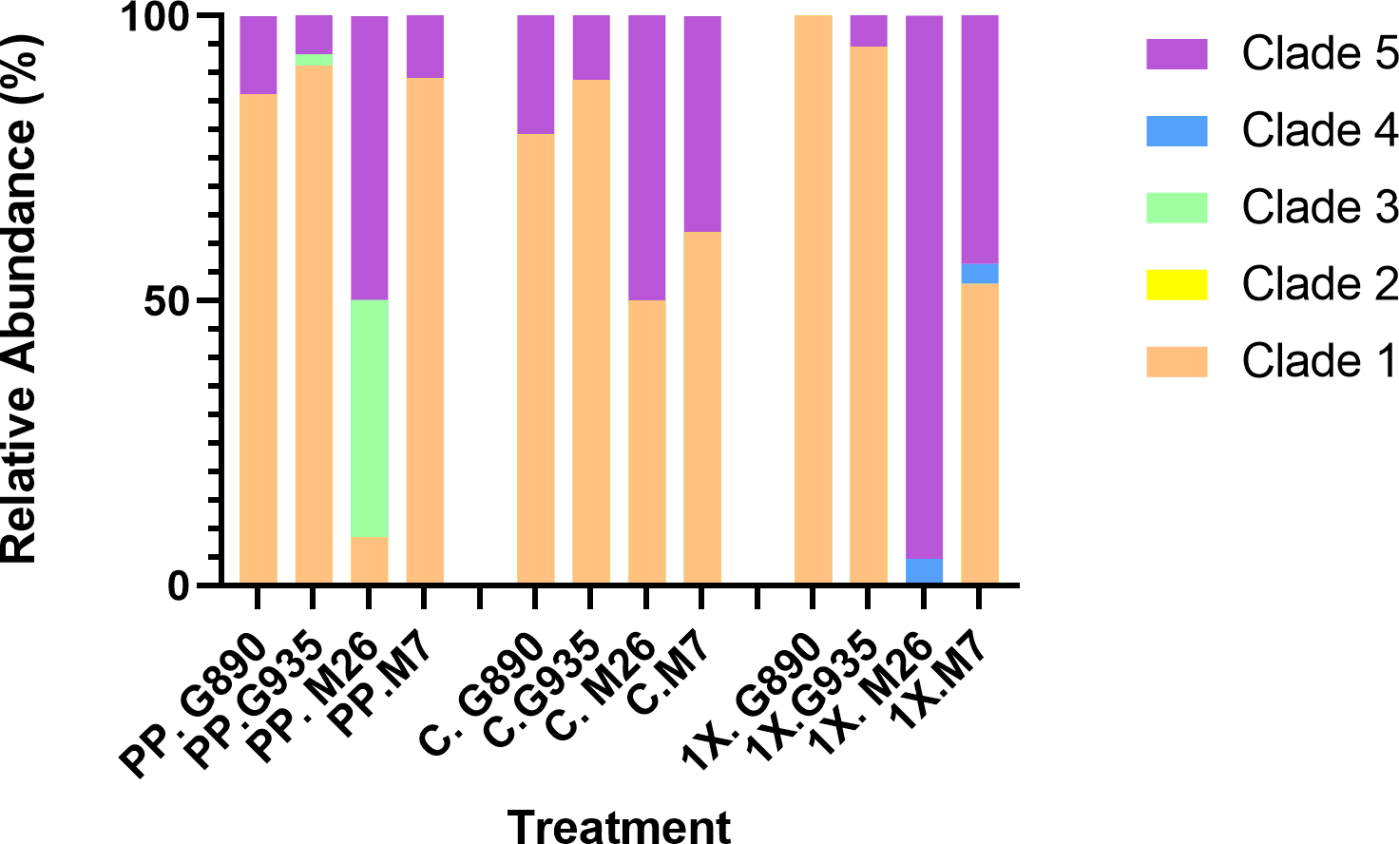
Stacked bar graph showing the percent relative abundance of different AMF taxa identified in the root tissue of four different rootstock genotypes pre-planting (PP), after cultivation in pasteurized control soil (C), and after cultivation in pasteurized soil inoculated with a commercial AMF consortia (1× CM) for 4 weeks. Taxonomic groups (clades) containing ASVs identified in this study are represented by different colors: orange (clade 1): *Rhizophagus* spp., yellow (clade 2): *Funneliformis* spp., green (clade 3): unknown sp., blue (clade 4): *Claroideoglomus* spp., purple (clade 5): Archaeosporales.

No Glomermycota spp. were identified in pasteurized soil, indicating that AMF were not present in the growth media prior to planting. All rootstocks were obtained from the same nursery location; however, prior to planting, significant differences in AMF community composition were identified between M.26 and M.7 (*P* = 0.004), M.26 and G.935 (*P* = 0.02), and M.7 and G.890 (*P* = 0.04) (Fig. 2). The AMF taxa detected in apple roots at this time likely represented those naturally occurring in nursery orchard soil and included those in clade 1 (*Rhizophagus* spp.), clade 3 (unknown), and clade 5 (Archaeosporales), but not clade 2 (*Funneliformis* spp.) or 4 (*Claroideoglomus* spp.) (Fig. 1). Of these taxa, *Rhizophagus* spp. (including *R. irregularis, R. fasciculatus*, and *R. vesiculiferus*) represented 91%, 89%, and 86% of the reads in G.935, M.7, and G.890, respectively, but only 8% of the reads in M.26 (Fig. 3).

In M.26, the nursery-derived AMF community was largely dominated by ASVs in clades 3 (40%) and 5 (50%) (Fig. 3). Phylogenetic analysis showed that clade 3 represented a unique, well-supported, monophyletic clade of unknown taxonomy (Fig. 1; Fig. S1). ASVs belonging to clade 3 were also detected in G.935 pre-planting, but at a much lower relative abundance (2%) than in M.26 (Fig. 1 and 3). Comparison of ASV sequences with those deposited in the NCBI 18S rRNA sequence (SSU) database (blastn) identified *F. mosseae* as the closest match (≤92% identity for ASVs 23, 85, 19, 49, 41, and 139); however, it is clear that clade 3 represents a distinct phylogenetic group with unique sequences that are not shared with the sequences from the literature in the alignment (Fig. 1). It should also be noted that, upon closer inspection, the high quality of the sequence data strengthens our confidence that these are unique sequences rather than artifacts.

ASVs belonging to clade 5 shared the highest similarity with *Ambispora* and *Archaeospora* (which represent sister taxa in our tree; Fig. S1) but contained extra, unique nucleotides in the sequence alignment (Fig. S2B). Upon close inspection, it was determined that these were not artifacts, so we did not trim these regions. Therefore, clade 5 ASVs formed a divergent group of sequences residing on long branches (Fig. 1). The large number of ASVs in clade 5 (Order Archaeosporales) likely represents a limited (albeit undetermined) number of AMF species. ASVs from clade 5 were present in all rootstock genotypes prior to planting but represented a much smaller percentage in G890 (14%), M7 (11%), and G.935 (7%) than in M.26 (50%).

### AMF detected in the commercial inoculum differed greatly from the product description

A total of 25 AMF amplicon sequence variants (ASVs) representing three different genera were detected in the DNA directly extracted from the commercial mixture. Phylogeny-based classification revealed that all AMF sequences detected in the CM samples either branched from clade 2 (*Funneliformis/Glomus;* 93%), clade 4 (*Claroideoglomus*; 6%), or clade 1 (*Rhizophagus;* 1%) (Fig. S1). In the CM samples, clade 1 was represented by *R. irregularis* (ASV7) and *Rhizophagus* sp. (ASV 174). ASVs belonging to clade 2 were most closely related to either *Funneliformis mosseae or Funneliformis coronatus*. One of the AMF species purported to be contained in the commercial mixture was *F. monosporum* (=*Glomus monosporum*, *F. mosseae;* https://invam.ku.edu/mosseae). Therefore, tree-based taxonomic assignments provided evidence that *F. mosseae* was in fact present in the mixture. As noted above, this AMF species was only detected in the commercial inoculum and did not appear to successfully colonize apple roots. This result was unexpected considering that *Funneliformis* species, including *F. mosseae*, have been identified in the apple orchards of Washington State and Italy (40, 57). No other AMF species, which were purported to be in the commercial product, were identified (Table 1). It should be noted that *Rhizophagus clarum* was purported to be in the commercial mixture, but this species was not detected in the CM samples. Overall, the commercial mixture contained a very different consortium of AMF than expected.

**TABLE 1:** AMF species purported to be in the commercial mixture vs those detected in the commercial mixture and in root tissue

AMF *purported* to be in the commercial mixture	Detected in commercial mixture
*Glomus clarum* (= *G. clarus, Rhizoglomus clarum*, *R. clarus*)	No
*Funneliformis monosporum (= F. mosseae, Glomus monosporum*)	Yes
*Septoglomus deserticola* (= *Glomus deserticola*)	No
*Paraglomus brasilianum* (= *Glomus brasilianum*)	No
*Gigaspora margarita*	No

AMF *detected* in the commercial mixture	Detected in apple root tissue
*Funneliformis mosseae / F. coronatus*	No
*Claroideoglomus luteum / C. claroideum*	Yes (M.26^*a*^ & M.7^*a*^)
*Claroideoglomus claroideum*	Yes (M.26^*a*^)
*Rhizophagus irregularis*	Yes (G.890^*b*^; G935^*b*^)

^*a*^ Detected in inoculated (1× Commercial Mixture) samples only.

^*b*^ Detected in inoculated and non-inoculated control samples.

### Apple rootstocks established relationships with introduced AMF in a genotype-specific manner

ASVs in clade 2 or 4 were not detected pre-planting or in plants cultivated in the no-AMF control soils and were considered to represent AMF introduced by the commercial mixture. *Claroideoglomus* species (clade 4) identified in the commercial inoculum successfully colonized both Malling rootstocks. For example, ASV 32, most closely related to *C. luteum*/*C. claroideum,* was detected in the CM and also in M.26 and M.7 (1× CM) samples (Fig. 1). In addition, ASVs 79 and 96 (most closely related to *C. claroideum*) were present in the commercial mixture and in M.26 (1× CM) samples (Fig. 1). Together, these three ASVs represented 5% and 3.5% of the AMF community in M.26 and M.7 (1× CM) treatments, respectively. By comparison, no *Claroideoglomus* ASVs were detected in the Geneva rootstocks used in this study (G.890 and G.935). It should, however, be noted that rootstock/AMF association patterns do not necessarily differentiate according to rootstock type (e.g., Geneva vs Malling).

### Introduced AMF may disproportionately affect low-abundance AMF taxa

Apple rootstock genotype appears to be an important factor influencing the assembly of AM fungal communities. In this study, the commercial mixture appeared to have a particularly large effect on M.26, especially its ability to maintain pre-established associations with *Rhizophagus* spp. Overall, *Rhizophagus* spp. only represented 10% of the initial (pre-established) AMF community in M.26. *Rhizophagus* spp. became more prominent when M.26 was cultivated in non-inoculated soil (50% relative abundance), but disappeared completely in the presence of the commercial inoculum (Fig. 3). One of the most dominant taxa, ASV 1 (*R. irregularis* or *R. vesciculiferous*)**,** was present in the nursery-derived root tissue of G.935 (48%), M.7 (45%), and G.890 (14%), but was undetectable in M.26 prior to planting. The relative abundance of ASV1 increased to 11.5% when M.26 was cultivated in no-AMF control soil, but was not detected in 1× CM treatments. This result suggests that ASV1 was present in M.26 root tissue prior to planting but only increased to detectable levels after planting in the absence of the commercial inoculum. In G.935 and M.7 rootstocks, however, the relative abundance of ASV1 remained relatively high (≥30%), regardless of treatment (Control or 1× CM), illustrating the stability of these particular AMF–rootstock associations. It is also interesting to note that, according to one-way ANOSIM analysis, G.935 and M.7 did not differ significantly in AMF community composition in any treatment (Fig. 2), a result which, in part, was due to their continued association with ASV 1. Taken together, these results suggest that M.26 may have been more susceptible to the loss of clade one taxa than other rootstock genotypes due to low initial (pre-plant) abundance.

Along these same lines, ASV2 (*R. fasciculatus*) was the most abundant AMF taxa (34%) identified in G.890 pre-planting, and this association remained relatively stable after planting (23%–26%), regardless of treatment. ASV2 was also detected in M.26 (7%) and M.7 pre-planting (3%), but these associations were not maintained in the presence of the commercial inoculum. By comparison, the relative abundance of ASV two in M.26 increased to 25% in non-inoculated control soil. One-way ANOSIM analysis also reflected the differential responses by M.26 and G.890 to the 1× CM treatment (but not to the control) (Fig. 2). The colonization of M.26 by introduced *Claroideoglomus* spp. may have contributed to the suppression/exclusion of resident *Rhizophagus* taxa, even though the abundance of *Claroideoglomus* spp. in M.26 was relatively low (5%) at the time of sampling. In M.7, colonization by *Claroideoglomus* sp. (ASV 32; 3.5%) may have also contributed to a slight reduction in the relative abundance of *Rhizophagus* spp. (no-AMF control = 62%; 1× CM = 53%). It is also worth noting that antagonistic interactions between *R. irregularis* and *C. claroideum* have been previously documented (58).

### Introduced AMF can influence resident AMF community structure regardless of colonization success

This finding is best is illustrated using clade 5 (Archaeosporales). Within this clade, ASVs 3, 4, and 11 (all terminal branches connected to a single node) most likely represent sequence variation within a single species (Fig. S1). These ASVs were detected in root tissue prior to planting in all rootstock genotypes (~1%–9%) and became enriched in all rootstock genotypes following cultivation in the no-AMF control soil (11%–35% relative abundance) (Fig. 1). In comparison, this same group of ASVs (3, 4, and 11) became suppressed in both Geneva rootstocks following cultivation in inoculated soil. This was surprising considering that no ASVs associated with the CM were detected in the Geneva rootstocks used in this study. In G.935, ASVs 3, 4, and 11 persisted in the presence of the commercial inoculum at lower relative abundance (5%) than in the control soil (11%). In G.890, however, not a single clade 5 ASV was detected in the 1× CM treatment, even though this group represented 21% of the AMF community when cultivated in the no-AMF control soil. The complete loss of clade 5 taxa from G.890 in 1× CM samples resulted in an increased dominance of clade 1 (*Rhizophagus* spp.), which comprised 100% of the AMF community (Fig. 3). This result suggests that less efficient AMF can alter or even displace ecologically relevant (indigenous) AMF communities living in symbiosis with the host plant, even if colonization by the introduced inoculant is not effective.

Although the commercial mixture appeared to negatively affect the ability of Geneva rootstocks to maintain relationships with indigenous ASVs in clade 5, it had a positive effect on their relative abundance in both Malling rootstocks. In M.26 and M.7, ASVs 3, 4, and 11 greatly increased in the presence of the commercial inoculum, representing 63% and 41% of the total reads (Fig. 3). This may be one reason why ordination analysis of relative abundance data indicated that although AMF communities in M.26 and M.7 were significantly different pre-planting (*P* = 0.004), they did not differ in the 1× CM treatment (Fig. 2). This result suggests that the introduced inoculant stimulated distantly related resident AMF species, an effect that is occasionally observed in studies with commercial inoculants (38). That said, the effects of the AMF inoculant on resident AMF in clade 5 were highly varied in M.26. Although ASVs 3, 4, and 11 increased in dominance in the 1× CM treatment (as described above), other clade 5 taxa disappeared. For example, a group of closely related sequences likely to represent a single species (ASVs 14, 28, and 16) accounted for 34% of the total AMF community when M.26 was cultivated in no-AMF control soil, but was not detected in the presence of the commercial inoculum (1× M.26). This result highlights the differential effects of the commercial inoculum on closely related AMF species within a particular rootstock. It is also worth noting that treatment was a significant source of variation affecting the absolute abundance of AMF, (Two-way ANOVA; *P* = 0.0001), which was generally higher prior to planting than after planting (Fig. S6). This reduction was most likely due to high levels of soil phosphorus (186 mg/kg) in potting soil. However, in all rootstock genotypes (except G.890), AMF abundance was further reduced in the presence of the commercial inoculant.

### Challenges associated with fine-scale differentiation between AMF species

It is important to note that genetically different nuclei can coexist within individual AMF spores (i.e., there is high within-spore variation in rRNA gene sequences) (59–61). In general, intraspecific sequence variability for *Rhizophagus* (and *R. irregularis* in particular) is known to be relatively high (62, 63). In this study, 27 different ASVs were associated with only three species of *Rhizophagus* (Fig. 1; clade 1). In Glomeromycotan taxa with high intrasporal rRNA sequence variation, sequences from different spores (or even different soil samples) can sometimes be more similar than sequences of the same spore or soil sample (64). In this study, ASV 7 (*R. irregularis;* clade 1) was not detected in Malling or Geneva rootstocks prior to planting but became enriched in Geneva rootstocks in both 1× and no-AMF control treatments; 1× G.890 (13%), 1× G.935 (12%), C.G890 (15%), and C.G935 (5%). ASV 7 was also detected in the commercial mixture. However, even though ASV or OTU-delimiting approaches yield the most biologically relevant taxonomic units for understanding AMF community composition, fine-scale, intrasporal genetic variation makes it difficult to tell whether ASV 7 originated solely from the commercial mixture.

### Plant growth responses

**Fig 4:**
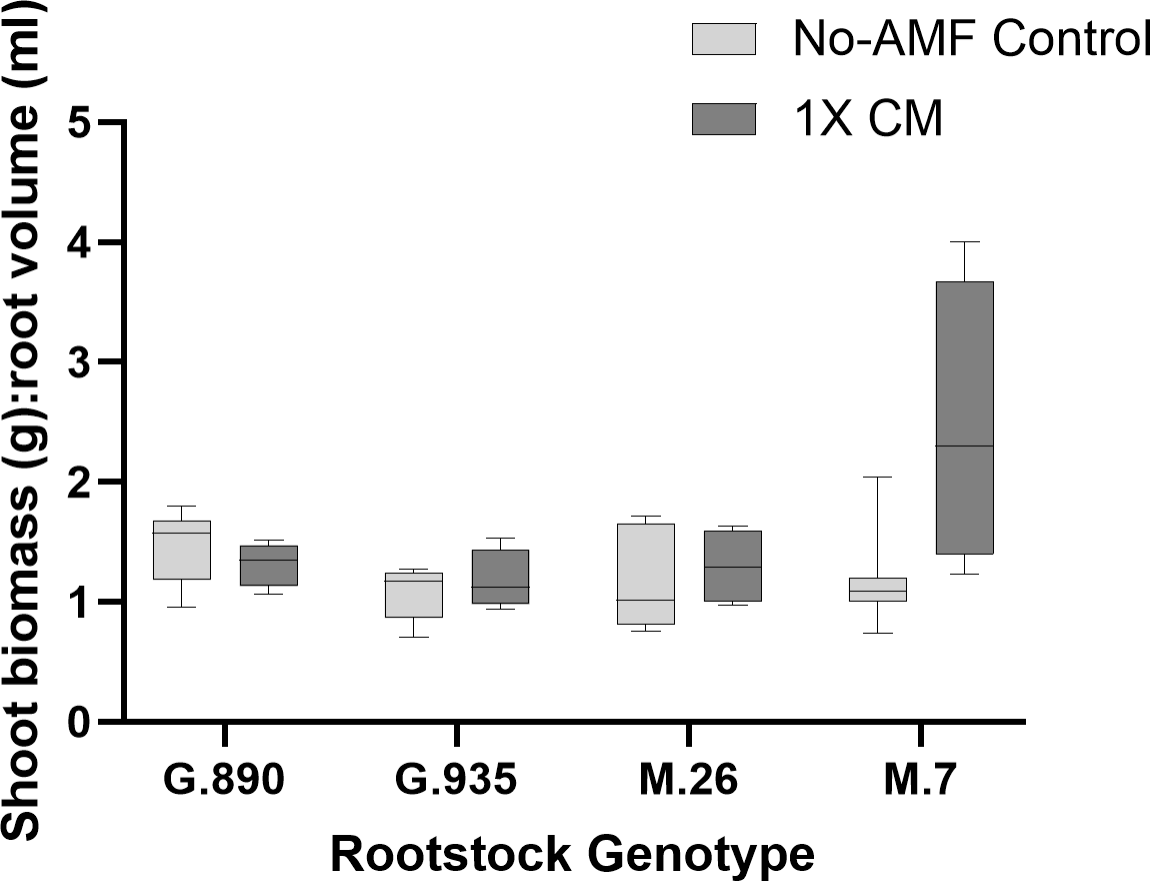
Ratio of shoot biomass: root volume measured after 4 weeks in apple rootstocks cultivated in pasteurized control soil (No-AMF Control; light gray) or pasteurized soil inoculated with a commercial AMF consortia (1× CM; dark grey).

The effect of the commercial AMF inoculant on resident AMF communities was also reflected in plant growth data. No significant differences in trunk diameter or shoot biomass were identified between inoculated and no-AMF control treatments. However, the root volume of M.7 plants cultivated in the inoculated (1× CM) soil was significantly reduced relative to those cultivated in the control treatment (*P* = 0.0008). The cause of the reduced root volume in M.7 plants cultivated in the 1× CM treatment is not clear as colonization of M.7 plants by the commercial inoculant *Claroideoglomus* sp. (ASV 32; 3.5%) did not appear to displace resident taxa or largely impact AMF community structure relative to no-AMF controls. ITS-based sequencing indicated that the fungal replant pathogens *Ilyonectria robusta* and *Rhizoctonia solani* (anastomosis groups unknown) were present in the root tissue of all rootstock genotypes, regardless of treatment. However, no significant differences in the relative abundance of either organism were identified between any of the rootstock genotypes in any treatment (one-way ANOVA; *P* < 0.05). It is possible that the commercial AMF consortia differentially impacted resource allocation relative to no-AMF controls in M.7; shoot:root ratios were close to being significantly different (Fig. 4; p_adj_ = 0.07; *P* = 0.01). Interactions between AMF and host plants have been shown to cause shifts in partitioning of biomass between shoots and roots in other studies (58).

## DISCUSSION

Over the past few years, interest in the use of products of microbial origin to promote sustainability in agricultural ecosystems has been growing. This includes interest in AMF inoculants, particularly in light of the increasing emphasis on soil health. The development of systematic and sustainable approaches for engineering the phytobiome as a means to improve crop production has even been referred to as “the next green revolution.” As part of this movement, the global agricultural biologicals market has seen rapid growth (65, 66). Despite this enthusiasm, however, a lack of understanding surrounding the modes of action of these products and the associated increases in plant yield or performance persists (65).

As referenced previously, the product used in this experiment was specifically selected because the manufacturer indicated it contained a diverse mix of AMF species. However, the product did not contain what it was purported to contain. This highlights the problem regarding the identification of AMF species identities contained in commercially available products. While this is concerning, it is not surprising, given the issues associated with the use of web-based databases for determining AMF identity (in our study, it was not essential to the experiment that the inoculum consist of the particular species purported to be contained in the mixture).

Obtaining accurate taxonomic assignments of DNA sequences from Phylum Glomeromycota is challenging (44). The standard “barcode” for molecular identification of fungi (the nuclear ribosomal internal transcribed spacer) does not provide adequate resolution of AMF taxa. Instead, the scientific community generally sequences the V3–V4 regions of the 18S rRNA gene (43, 44). At present, there are no actively curated (i.e., up to date) web-based 18S rRNA databases. Therefore, in order to describe arbuscular mycorrhizal fungal communities at the genus and species-level, sequence data must be placed onto an AMF-specific phylogenetic tree. This is an area of research that needs attention because it has important ramifications for the successful application of AMF inoculants. The use of phylogenetic-based tools by product developers and industry practitioners would ensure quality-control in terms of AMF product composition/consistency.

Although taxonomic assignment can be greatly improved using phylogenetics, it should be noted that accurate identification of AMF based on nuclear ribosomal DNA remains a challenge. The available molecular data (i.e., rDNA reference sequences with sufficient length) is not yet complete in terms of the diversity of taxon coverage. Furthermore, less than half of the 237 currently described AMF species are propagated by INVAM (https://invam.ku.edu/species-diversity). This is partly due to the obligate biotrophic nature of AMF as well as their morphological plasticity, traits which make characterizing biological material from described species difficult. Due to the paucity of data on the interactions of commercial AMF inoculants with established indigenous AMF communities, one of the primary goals of this study was to establish whether or not apple rootstocks serve as a significant source of AMF inoculum from the nursery where they are produced. In general, pre-established taxa would be expected to be favored over introduced species due to priority effects (67). Thus, we hypothesized that nursery-derived AMF would strongly influence AMF community structure after planting and limit effective colonization by mycorrhizal inoculants. In order to further explore the selective capacity of host genotype on the root-associated AMF community, a diverse group of apple rootstock genotypes was included. The resulting data showed that the ability of a commercial inoculant to effectively alter and/or compete with pre-existing AMF largely depends on the AMF species/rootstock present. In this study, the *Claroideoglomus* species (*C. claroideum* and *C. luteum*) contained in the commercial mixture were able to successfully compete with the resident AMF populations contained in Malling but not Geneva rootstocks. Therefore, the ability of an apple plant to maintain its resident AMF community likely depends on rootstock-specific filters, which influence the composition of the species pool. The findings from this study also suggest that strength of the priority effect may depend on the relative abundance of the resident species. For example, although *Rhizophagus* disappeared completely when M.26 was cultivated in inoculated soil, the group only represented only 10% of the community to begin with (compared to >86% in all other rootstocks). Low-abundant AMF taxa were also found to be prone to disappearance in a study that assessed the effects of agricultural management intensity levels on AMF community assembly (68). Rare taxa play pivotal roles in maintaining the stability of plant-associated microbiomes. In fact, Xiong et al. showed that “hub” species of fungal co-occurrence networks in rhizosphere soil and root tissue were primarily comprised of rare taxa, including AMF (69). Rare taxa also contribute to community stability by acting as a reservoir that can rapidly respond to environmental change (70). Hence, commercially available AMF products have the potential to alter the resident AMF community in ways that negatively impact the entire plant-associated microbiome.

Regardless of rootstock genotype, nursery-established AMF communities did appear to strongly limit the plant’s ability to interact with introduced AMF propagules, suggesting that rootstocks serve as a significant source of AMF inoculum from nurseries where they are produced. For example, as previously mentioned, *F. mosseae* is considered to have a high aptitude for colonization with apple. Therefore, it was surprising that the introduced *Funneliformis* spp. were unable to infect any of the plants within the experimental timeframe (4 weeks)*,* especially considering that *Funneliformis* spp. (clade 2) represented 93% of the AMF contained in the inoculum, while clade 4 (*Claroideoglomus*) represented a much lower percentage (6%). In addition, a number of different rootstock/AMF interactions that were established pre-planting persisted, regardless of treatment. These included G.935/M.7 × *R. irregularis/R. vesciculiferous,* G.890 × *Rhizophagus fasciculatus* as well as ASVs 3, 4, and 11 in M.26 and M.7. These results highlight the potential for nursery-established AMF associations to be maintained when transplanted into the field.

On the other hand, the data showed that AMF inoculants do not necessarily need to outcompete the existing resident AM fungal community to have an effect on resident AMF communities living in symbiosis with the host plant and/or plant growth. For example, although AMF present in the CM did not effectively colonize the Geneva rootstocks used in this study, the effect of inoculation on AMF community dynamics was evidenced in other ways. The most dramatic example was the complete loss of clade 5 from G.890, even though this group increased from 14% to 21% of the AMF community when cultivated in the no-AMF control soil. These results are mirrored by another study in which inoculated AMF taxa failed to colonize maize plant roots but altered species dominance and reduced diversity of the pre-existing AMF community (71).

Predicting AMF community dynamics in apple rootstocks is complex (even in an AMF-free growth medium). In conventional nursery systems, rootstocks are typically propagated in stoolbeds (a process by which new shoots from a “mother tree” develop roots). In this way, relationships with AMF are naturally established and nursery-derived material is never “clean” to begin with. More recently, plant tissue culture has become a major propagation tool for fruit tree rootstocks and is expected to become the standard going forward as techniques continue to improve. This approach to rootstock propagation may present new opportunities to inoculate “AMF-free” plant material with ecologically relevant AMF species. In a study by Calvet et al., micropropagated “AMF-free” peach rootstocks inoculated with a mixed community of *Glomus* spp. prior to transplantation into orchard soil significantly reduced nematode populations in roots relative to non-inoculated plants (72).

Manipulating AMF community re-assembly post-fumigation is another potential path forward. Broad spectrum soil fumigation significantly reduces pathogen activity (and improves tree growth) but also greatly depresses the entire soil microbiome (including native AMF communities). The loss of the resident microbial population impairs the ability of the soil to defend against re-infestation. However, if applied to recently fumigated soil, AMF taxa (e.g., *Claroideoglomus* spp.) could potentially prevent the re-infestation of later-arriving pathogenic organisms *via* priority effects. In a study by Resendes et al., once an apple root was colonized by AMF, it was no longer accessible to a potential pathogen (and *vice versa*) (73). Therefore, in the context of orchard/nursery management, introduced AMF must be capable of establishing in new root tissue in a short period of time in order to compete with soilborne root pathogens (and other indigenous microbes already present). Moreover, the fastest AMF colonizers are also often the most extensive (74).

Since pasteurized soil is a proxy for fumigation, the results of the current study suggest that commercial AMF inoculants applied to recently fumigated soil can become established in new root tissue alongside AMF, which are pre-established at the nursery. That said, our study also highlights the importance of considering the dependency of AMF community structure in regard to rootstock genotype. It is not clear why the *Claroideoglomus* spp. contained in the commercial inoculum were unable to successfully colonize the Geneva rootstocks evaluated in this study. The differential colonization of rootstock genotypes by AMF contained in the commercial inoculum provides evidence for the selective capacity of host genotype on the plant-associated AMF microbiome. Few studies have documented the selective capacity of apple rootstock genotype on the endophytic microbiome, especially AMF assemblages (40, 41). In the study by Cook et al., both apple rootstock genotype and AMF species were found to be significant sources of variation affecting the percentage of colonization (41). However, significant interaction effects between these two factors were not identified. Among the AMF tested, *C. etunicatum* and *R. irregularis* represented the most compatible fungal partners in pasteurized orchard soil, regardless of apple rootstock genotype (41). In our study, nursery-derived communities were dominated by *Rhizophagus* spp. in three out of four of the rootstock genotypes tested.

It is also interesting to note that in the study by Van Horn et al., G.890 rootstocks consistently harbored the highest percentage of arbuscular mycorrhizal fungal species (>5% of the total endophytic fungal community) (40). In the current study, although genotype was not a significant source of variation affecting absolute abundance in any of the treatments (two-way ANOVA; *P* = 0.09), AMF DNA reached the highest levels in G.890 no-AMF control and 1× CM treatments (Fig. S6). These two (G.890) treatments also contained the most AMF in terms of relative abundance (3.3 and 5.7%, respectively). That said, because the initial nursery-derived AMF communities varied significantly between rootstock genotypes, our ability to explore the selective capacity of host genotype on the plant-associated AMF microbiome was limited.

Finally, the use of a Glomeromycota-specific phylogenetic tree was critical for our ability to accurately assign taxonomy to AMF sequences and supported the detection of a divergent group of sequences within the Order Archaeosporales, which were present in all rootstocks prior to planting. It is likely that a relatively small proportion of all existing Archaeosporales have been characterized (43). In addition, our phylogenetic tree supported the detection of a new, undescribed lineage of AMF (clade 3), possibly within the Order Glomerales. These results highlight the need for studies going forward to continue to utilize and broaden the molecular data available for phylogenetic-based classification of AMF communities. A better understanding of what “real” AMF communities look like is an essential component to the successful transfer of compatible rootstock/AMF combinations from the laboratory to field.

## Data Availability

The sequence data generated and analyzed during the current study are available in the NCBI SRA repository (BioProject PRJNA1124126). Any other data generated or analyzed during this study are included in this published article and its supplemental material or are available from the corresponding author upon reasonable request.

## SUPPLEMENTAL MATERIAL

File S1 aem.01937-24-s0001.txt Glomeromycota 18S rRNA reference sequences, based on Krueger et al., 2012.

File S2 aem.01937-24-s0002.txt Glomeromycota 18S rRNA reference sequences, based on Stefani et al.

Figure S1 aem.01937-24-s0003.pdf Phylogenetic tree inferred with Glomeromycotan ASV sequences from this experiment plus reference sequences from Krueger *et al.*, 2012.

Figure S2 aem.01937-24-s0004.pdf Sequence alignment of ASVs from this study and Krueger *et al.*, 2012.

Figures S3 and S4 aem.01937-24-s0005.docx Median read length of 18S rRNA Glomeromycotan sequence reads following cleaning/de-noising, and rarefaction analyses showing sequencing depth (reads per sample).

Figure S5 aem.01937-24-s0006.pdf Comparison of Glomeromycotan phylogenies from this study and two previously publish studies.

Figure S6 aem.01937-24-s0007.tif Amount of AMF DNA detected in root tissue as estimated from qPCR of total fungal DNA.

Supplemental legends aem.01937-24-s0008.docx Legends for Fig. S1 to S6.

Table S1 aem.01937-24-s0009.docx Number of reads remaining after each step in the sequence processing.

Table S2 aem.01937-24-s0010.xlsx Relative abundance of each ASV, calculated per the total number of Glomeromycota reads per sample.

ASM does not own the copyrights to Supplemental Material that may be linked to, or accessed through, an article. The authors have granted ASM a non-exclusive, world-wide license to publish the Supplemental Material files. Please contact the corresponding author directly for reuse.
