# Erratum for Zhang et al., “Evaluating the stability of nursery-established arbuscular mycorrhizal fungal associations in apple rootstocks”

**DOI:** 10.1128/aem.00198-25

**Published:** 2025-02-21

**Authors:** Huiting Zhang, Wanyan Wang, Loren Honaas, Mark Mazzola, Tracey Somera

## ERRATUM

Volume 91, no. 1, e01937-24, 2025, https://doi.org/10.1128/aem.01937-24. Loren Honaas’ name should appear as shown in this erratum.

