# Supplementary figures and images for: Evaluating the stability of nursery-established arbuscular mycorrhizal fungal associations in apple rootstocks
Source: Appl Environ Microbiol. 2024 Dec 10;91(1):e01937-24. doi: 10.1128/aem.01937-24 (PMC11784189; doi:10.1128/aem.01937-24)

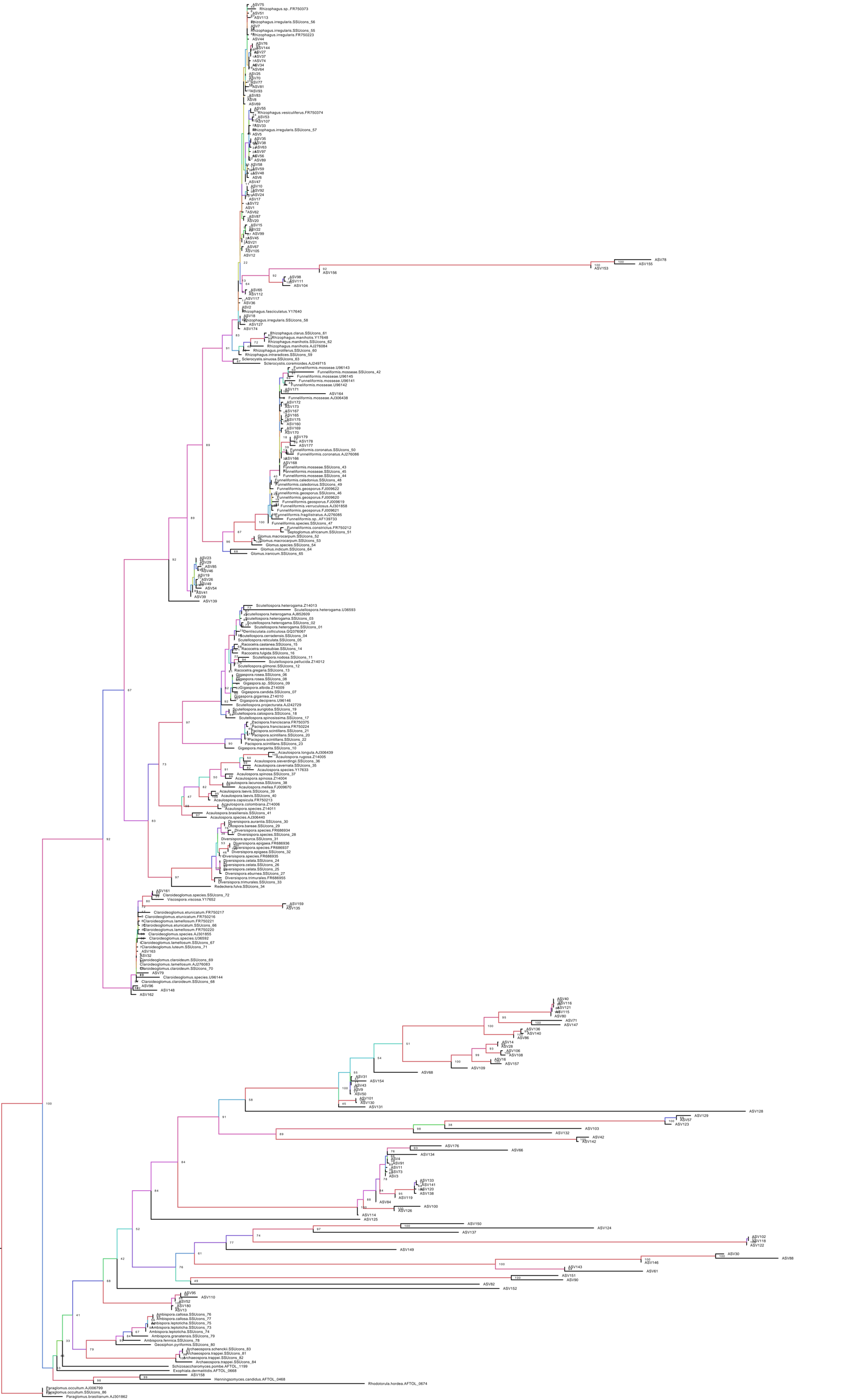

Supplement: Figure S1 — Phylogenetic tree inferred with Glomeromycotan ASV sequences from this experiment plus reference sequences from Krueger et al., 2012. [file aem.01937-24-s0003.pdf]

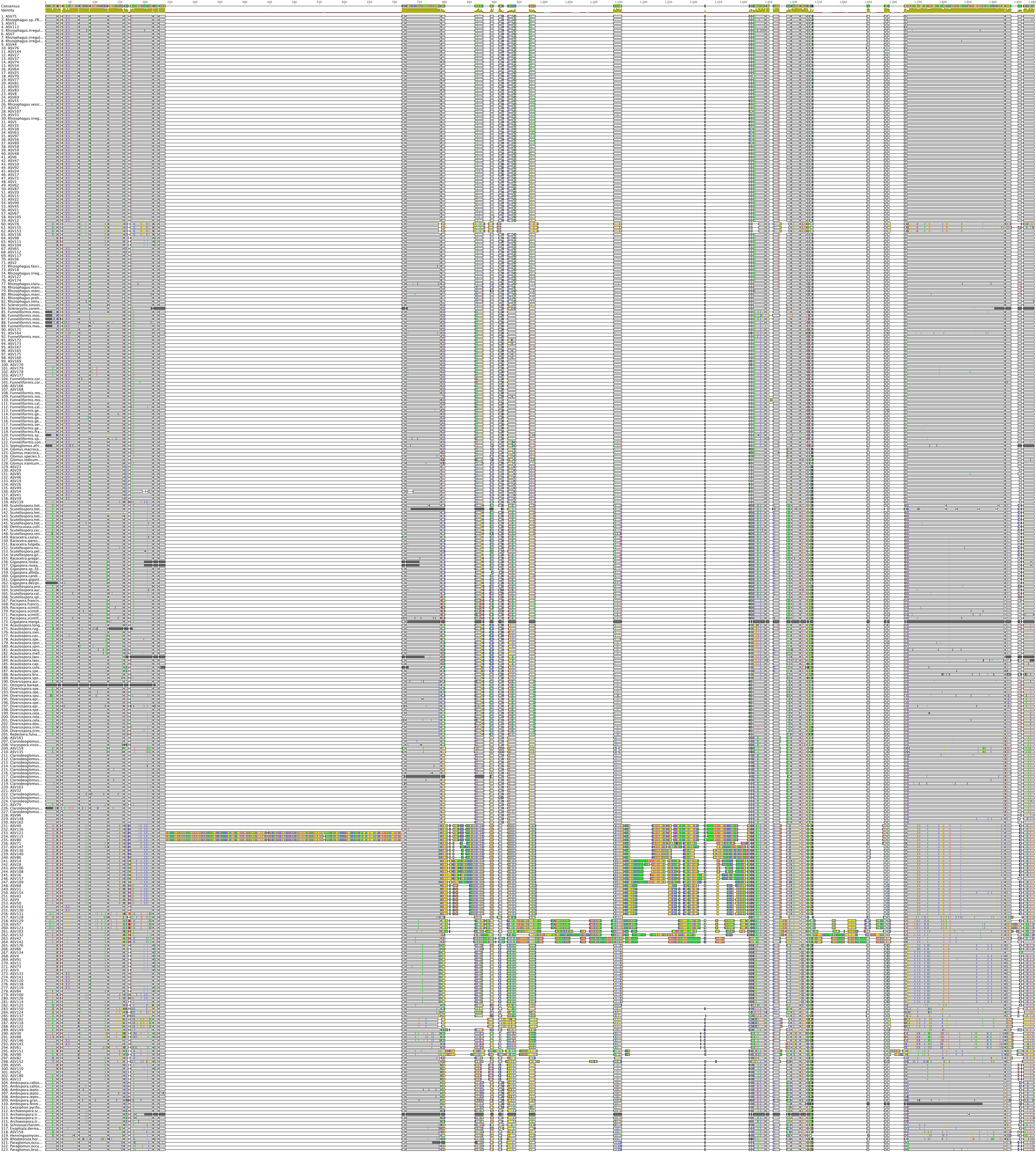

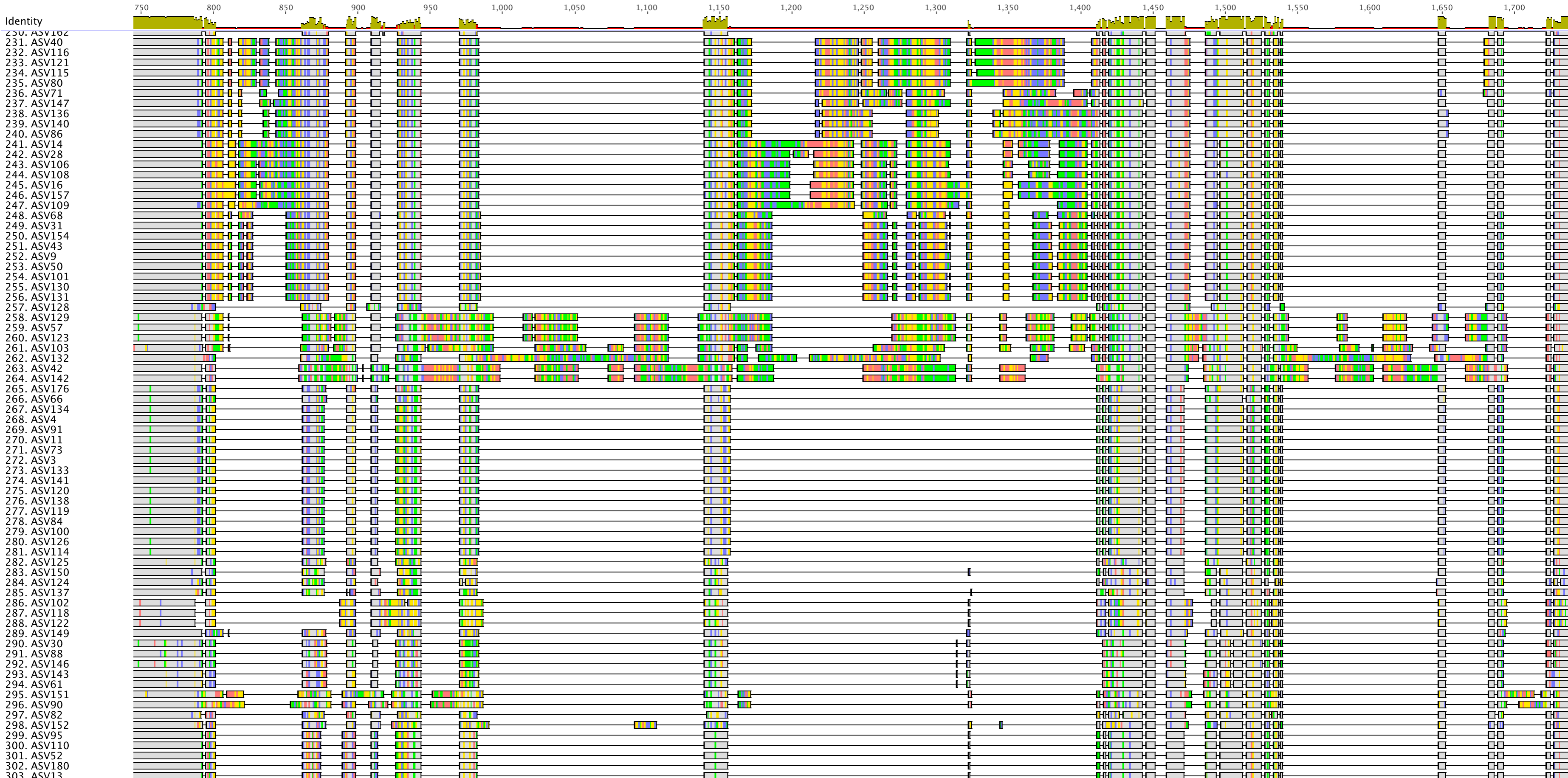

Supplement: Figure S2 — Sequence alignment of ASVs from this study and Krueger et al., 2012. [file aem.01937-24-s0004.pdf]

**A. This study**

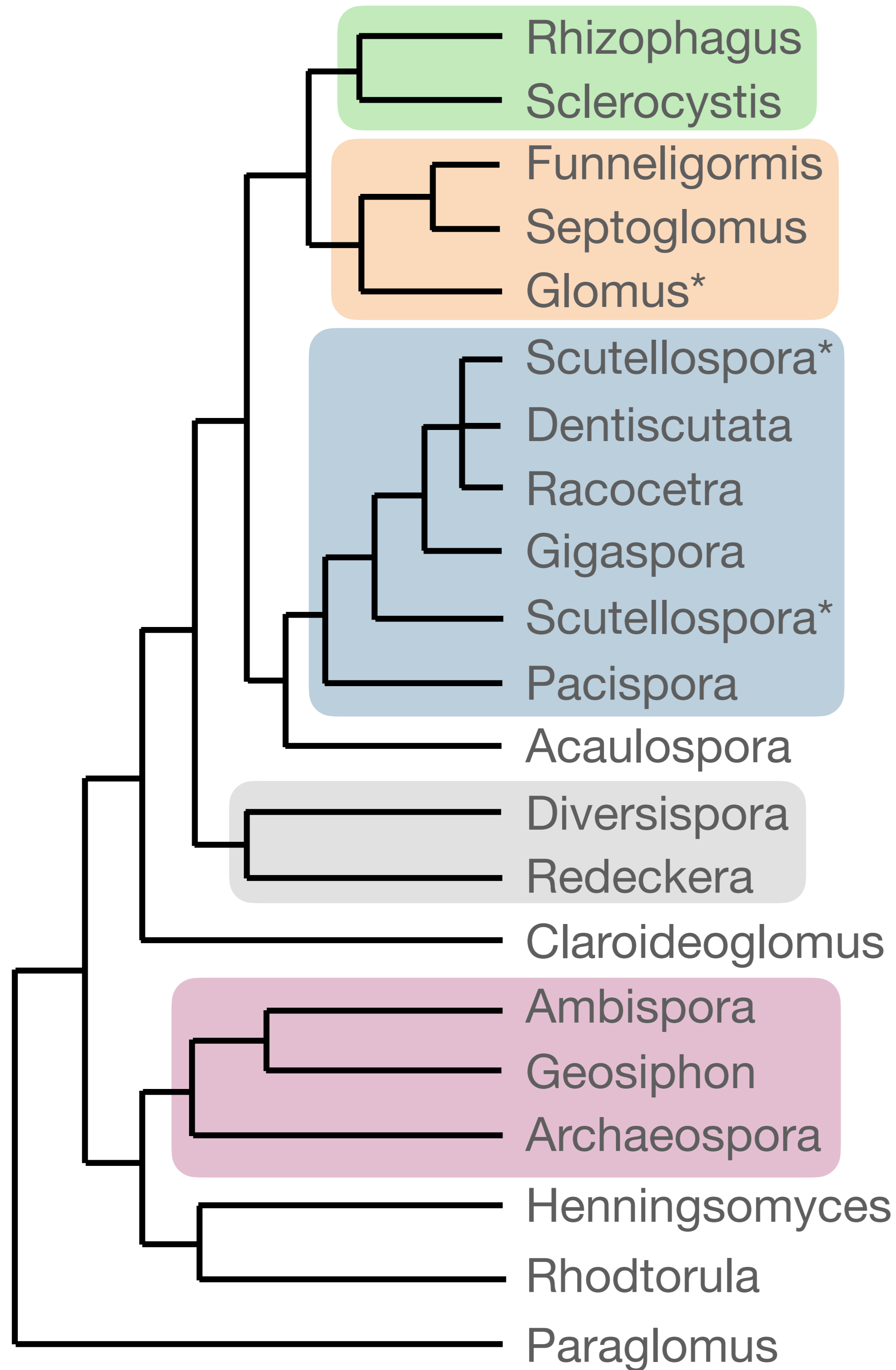

**B. Kruger et al., 2012**

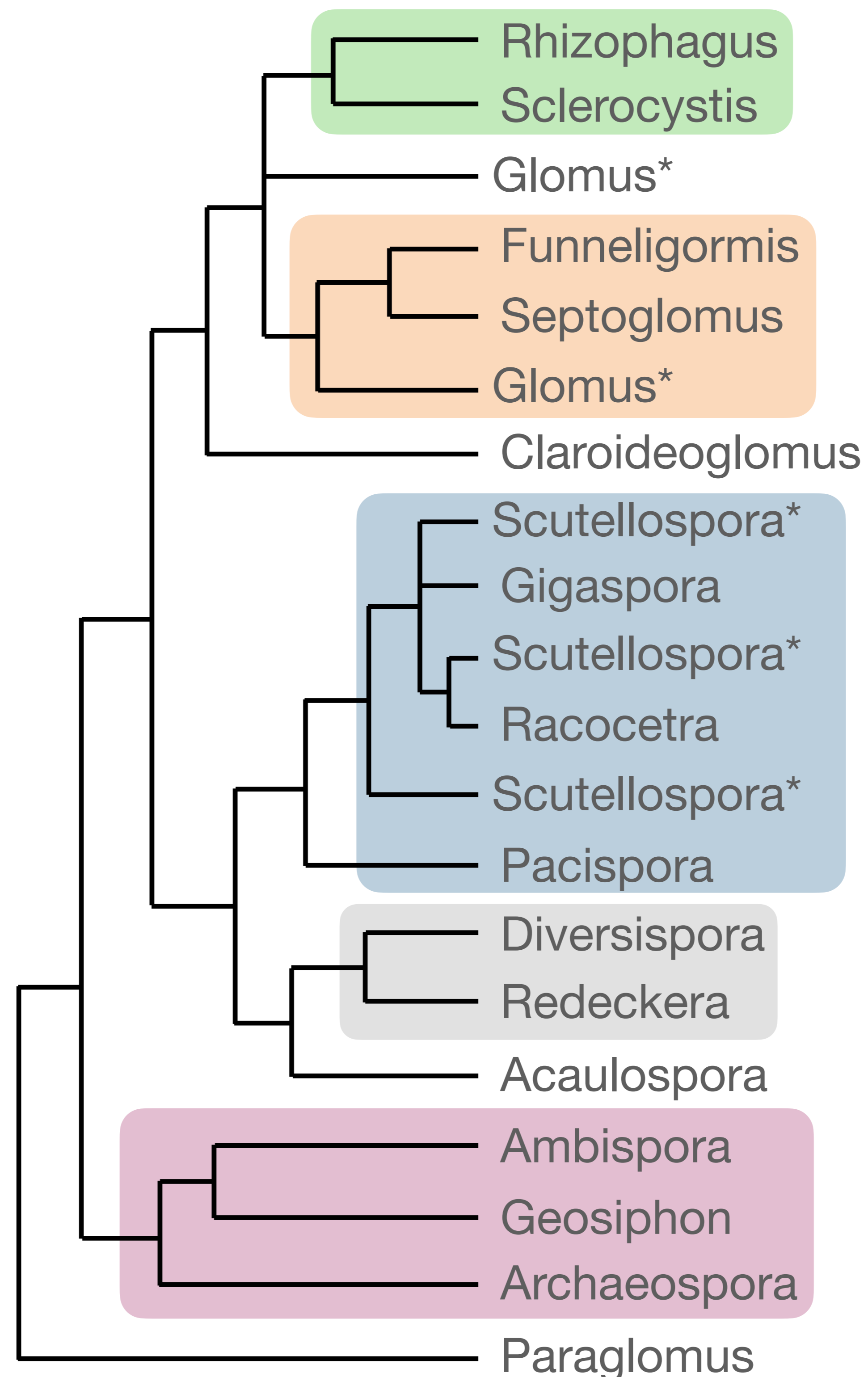

**C. Stefani et al., 2020**

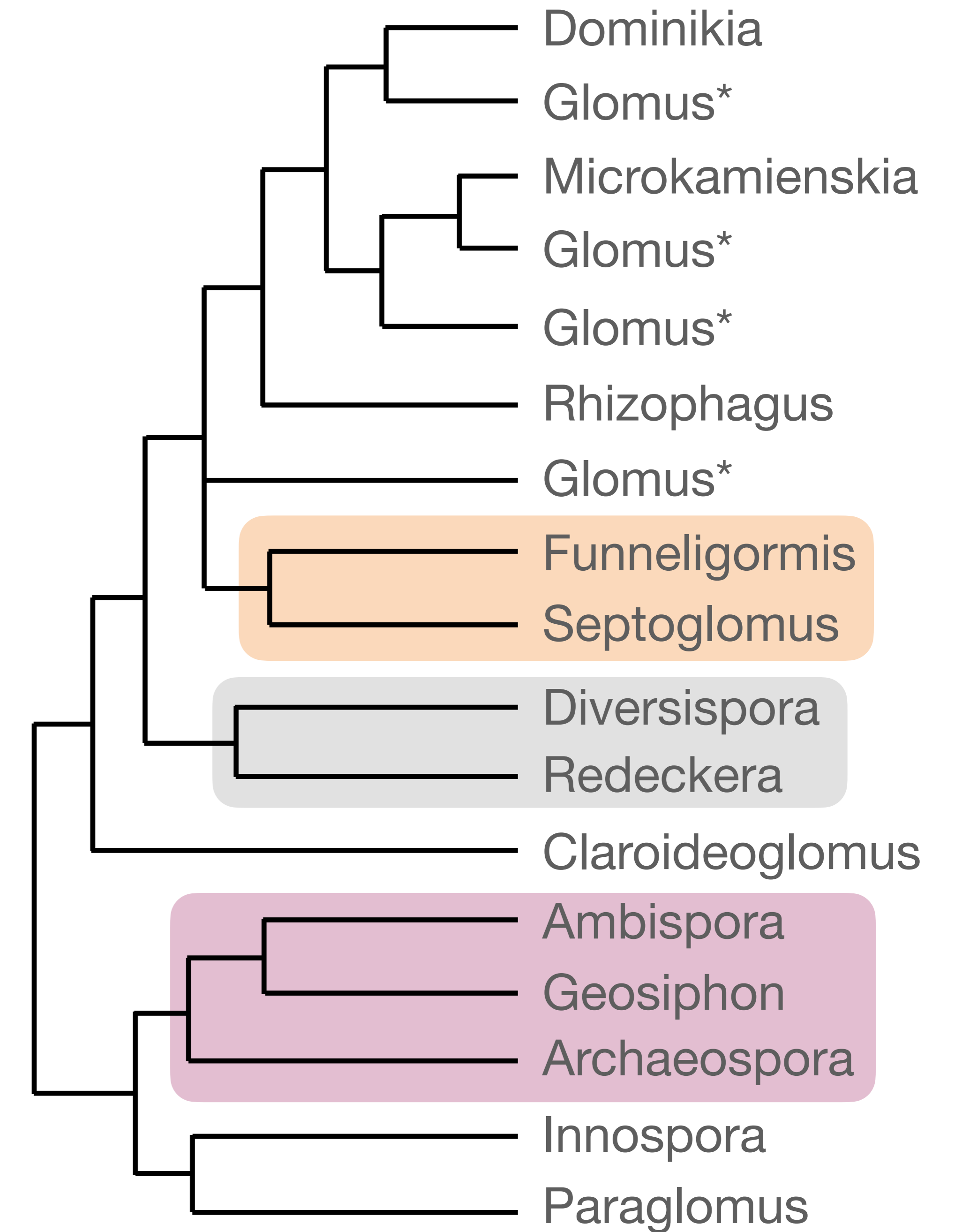

Supplement: Figure S5 — Comparison of Glomeromycotan phylogenies from this study and two previously publish studies. [file aem.01937-24-s0006.pdf]

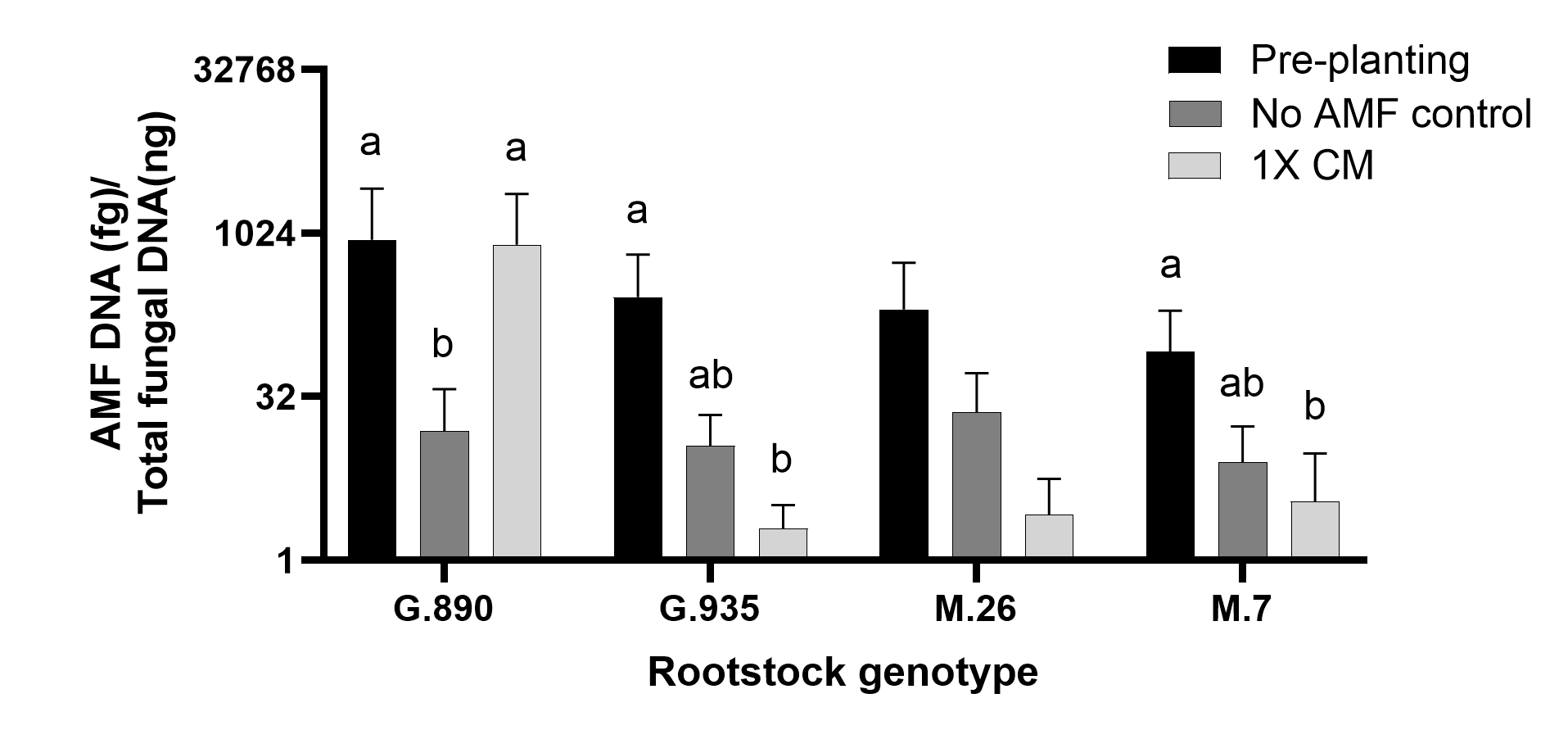

Supplement: Figure S6 — Amount of AMF DNA detected in root tissue as estimated from qPCR of total fungal DNA. [file aem.01937-24-s0007.tif]
