# Supplementary material for: Evaluating the stability of nursery-established arbuscular mycorrhizal fungal associations in apple rootstocks
Source: Appl Environ Microbiol. 2024 Dec 10;91(1):e01937-24. doi: 10.1128/aem.01937-24 (PMC11784189; doi:10.1128/aem.01937-24)
Supplement: Figures S3 and S4 — Median read length of 18S rRNA Glomeromycotan sequence reads following cleaning/de-noising, and rarefaction analyses showing sequencing depth (reads per sample). [file aem.01937-24-s0005.docx]

**Figure S3**: Median read length of 18S rRNA Glomeromycotan sequence reads following cleaning/de-noising.


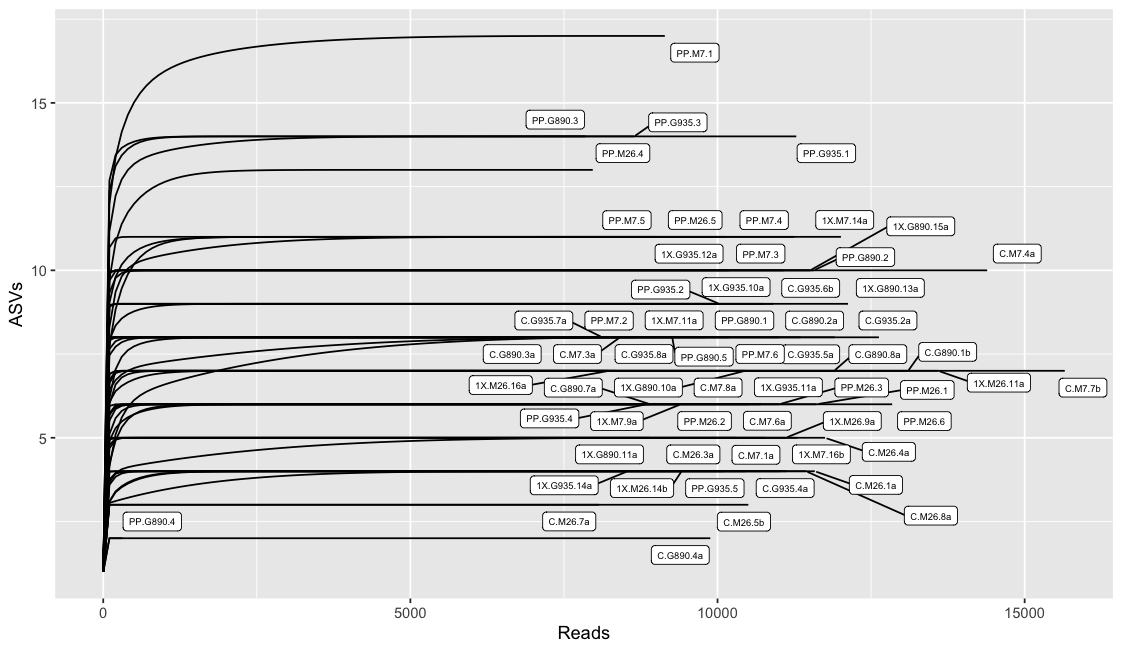


**Figure S4:** Rarefaction analyses showing sequencing depth (reads per sample).
