# Supplementary material for: Evaluating the stability of nursery-established arbuscular mycorrhizal fungal associations in apple rootstocks
Source: Appl Environ Microbiol. 2024 Dec 10;91(1):e01937-24. doi: 10.1128/aem.01937-24 (PMC11784189; doi:10.1128/aem.01937-24)
Supplement: Supplemental legends — Legends for Fig. S1 to S6. [file aem.01937-24-s0008.docx]

**Supplementary Figure S1.** Phylogenetic tree inferred with Glomeromycotan ASV sequences from this experiment plus reference sequences from Krueger et al, 2012.

**Supplementary Figure S2.** **Sequence alignment of ASVs from this study and Krueger et al., 2012.** **(A)** Alignment of all sequences used for phylogenetic tree inference. Sequences are arranged to follow the same order as in the phylogenetic tree in Supplementary Figure S1. **(B)** Close-up view of the highly divergent region of the clade 5 sequences in the alignment. In both A and B, each row represents a sequence and each column represents a site in the alignment. Sites colored in grey indicate a same nucleotide in a given sequence compared to the consensus; sites with a color (A: red; T: green; C: purple, G: yellow) indicate a difference compared to the consensus. Colors in the identity row indicate mean pairwise identity over all pairs in the column (army green: >30% and <100% identity; red: <30% identity).

**Supplementary Figure S3**: Median read length of 18S rRNA Glomeromycotan sequence reads following cleaning/de-noising.

**Supplementary Figure S4:** Rarefaction analyses showing sequencing depth (reads per sample).

**Supplementary Figure S5. Comparison of Glomeromycotan phylogenies from this study and two previously publish studies.** Simplified phylogenetic trees from **(A)** this study, **(B)** Krueger et al., 2012 with some branches rotated to match tree A, and **(C)** Stefani et al., 2020. Trees were simplified into cladograms with each node representing the major taxonomic group of a monophyletic group in the original tree. Only species information from the reference sequences were used to create the tree in (A). Monophyletic taxa groups shared between the three trees are highlighted with the same-colored background. Taxa with an ‘*’ appended after the name appeared at multiple locations in the tree, namely unresolved paraphyletic groups.

**Supplementary Figure S6. Amount of AMF DNA detected in root tissue as estimated from qPCR of total fungal DNA.** Values were normalized by log-transformation (y=log(y)) and two-way ANOVA followed by Tukey's multiple comparisons test was used to check for significant differences between treatments. Different letter groups indicate significant differences between treatments within each genotype; data are shown on a log_2_ scale.
