# Supplementary material for: Evaluating the stability of nursery-established arbuscular mycorrhizal fungal associations in apple rootstocks
Source: Appl Environ Microbiol. 2024 Dec 10;91(1):e01937-24. doi: 10.1128/aem.01937-24 (PMC11784189; doi:10.1128/aem.01937-24)
Supplement: Table S1 — Number of reads remaining after each step in the sequence processing. [file aem.01937-24-s0009.docx]

**Supplementary Table S1: The number of reads remaining after each step in the sequence processing.** Sequencing results were demultiplexed in house and then pre-processed using the DADA2 (v1.26.0) pipeline which included trimming, quality filtering and dereplicating. Dereplicated sequences were then used for denoising/chimera removal.

|  | **Demultiplexed** | **Primers removed** | **Quality-filtered** | **De-noised** | **Final # of unique seq** |
| --- | --- | --- | --- | --- | --- |
| 1X.G890.10a | 12583 | 10481 | 10456 | 10450 | 8242 |
| 1X.G890.11a | 10980 | 8972 | 8944 | 8905 | 7011 |
| 1X.G890.13a | 14596 | 12163 | 12133 | 12125 | 9654 |
| 1X.G890.15a | 13998 | 11566 | 11529 | 11509 | 9989 |
| 1X.G935.10a | 13123 | 10914 | 10869 | 10753 | 9076 |
| 1X.G935.11a | 13228 | 10829 | 10775 | 10705 | 8016 |
| 1X.G935.12a | 12332 | 10353 | 10307 | 10200 | 8293 |
| 1X.G935.14a | 10866 | 8903 | 8867 | 8536 | 7343 |
| 1X.M26.11a | 17198 | 14543 | 14465 | 13592 | 12274 |
| 1X.M26.14b | 11877 | 9824 | 9781 | 9415 | 8475 |
| 1X.M26.16a | 10672 | 8607 | 8569 | 8235 | 7438 |
| 1X.M26.9a | 14199 | 11749 | 11342 | 11111 | 8990 |
| 1X.M7.11a | 11992 | 10093 | 10065 | 9808 | 8453 |
| 1X.M7.14a | 16175 | 13149 | 13071 | 12009 | 12013 |
| 1X.M7.16b | 16681 | 13798 | 11451 | 11302 | 9175 |
| 1X.M7.9a | 11331 | 9557 | 9527 | 9402 | 7411 |
| C.G890.1b | 15655 | 13160 | 13117 | 13104 | 10086 |
| C.G890.2a | 14671 | 12072 | 12039 | 11905 | 9848 |
| C.G890.3a | 9940 | 7304 | 7284 | 7172 | 5807 |
| C.G890.4a | 12292 | 10176 | 10152 | 9882 | 7702 |
| C.G890.7a | 11211 | 9116 | 9076 | 8905 | 7783 |
| C.G890.8a | 14421 | 11997 | 11962 | 11899 | 9508 |
| C.G935.2a | 15586 | 12908 | 12851 | 12628 | 11309 |
| C.G935.4a | 13463 | 11184 | 11150 | 11125 | 8529 |
| C.G935.5a | 13987 | 11473 | 11434 | 11352 | 9814 |
| C.G935.6b | 13522 | 11042 | 10998 | 10911 | 9652 |
| C.G935.7a | 10040 | 8358 | 8328 | 8124 | 7462 |
| C.G935.8a | 10671 | 8691 | 8655 | 8346 | 7683 |
| C.M26.1a | 14120 | 11635 | 11603 | 11584 | 8496 |
| C.M26.3a | 12404 | 10196 | 10176 | 10103 | 7242 |
| C.M26.4a | 15783 | 12442 | 12346 | 11751 | 11241 |
| C.M26.5b | 12778 | 10613 | 10580 | 10502 | 7930 |
| C.M26.7a | 10062 | 8366 | 8324 | 8073 | 7708 |
| C.M26.8a | 14458 | 12182 | 12116 | 11442 | 11480 |
| C.M7.1a | 13235 | 11182 | 11146 | 11021 | 9245 |
| C.M7.3a | 10620 | 8849 | 8799 | 8413 | 7989 |
| C.M7.4a | 18172 | 14939 | 14880 | 14390 | 12511 |
| C.M7.6a | 13058 | 10901 | 10870 | 10775 | 8811 |
| C.M7.7b | 19931 | 16177 | 16093 | 15653 | 13783 |
| C.M7.8a | 12506 | 10614 | 10582 | 10512 | 8191 |
| PP.G890.1 | 13095 | 11049 | 11021 | 11004 | 7364 |
| PP.G890.2 | 14056 | 11641 | 11594 | 11567 | 9223 |
| PP.G890.3 | 11341 | 8543 | 8330 | 7850 | 7176 |
| PP.G890.4 | 477 | 321 | 318 | 311 | 233 |
| PP.G890.5 | 11387 | 9517 | 9467 | 9263 | 7737 |
| PP.G935.1 | 14836 | 12164 | 12110 | 11282 | 10600 |
| PP.G935.2 | 12132 | 10084 | 10051 | 10031 | 7835 |
| PP.G935.3 | 9979 | 8732 | 8700 | 8645 | 6695 |
| PP.G935.4 | 11066 | 9127 | 9026 | 8841 | 6424 |
| PP.G935.5 | 12488 | 10505 | 10474 | 10455 | 7295 |
| PP.M26.1 | 14161 | 11727 | 11639 | 11612 | 10213 |
| PP.M26.2 | 12514 | 10418 | 10326 | 10307 | 9369 |
| PP.M26.3 | 13334 | 11098 | 11056 | 11004 | 8630 |
| PP.M26.4 | 12348 | 9483 | 9276 | 7969 | 8302 |
| PP.M26.5 | 12587 | 10709 | 10675 | 10129 | 9118 |
| PP.M26.6 | 15711 | 13166 | 13116 | 12843 | 10568 |
| PP.M7.1 | 12690 | 9720 | 9663 | 9142 | 7782 |
| PP.M7.2 | 10610 | 8975 | 8941 | 8663 | 5207 |
| PP.M7.3 | 13319 | 11168 | 11121 | 11075 | 8981 |
| PP.M7.4 | 13079 | 11305 | 11278 | 11248 | 8077 |
| PP.M7.5 | 10679 | 9226 | 9200 | 9015 | 6099 |
| PP.M7.6 | 12060 | 10257 | 10228 | 10202 | 8160 |
